# Cerebrospinal fluid cytology findings in a case of diffuse leptomeningeal glioneuronal tumour

**DOI:** 10.1111/cyt.13169

**Published:** 2022-08-11

**Authors:** Melanie P. Jensen, Emma Lim, Luke Dixon, Benoit Quilichini, Harri Jenkins, Patrizia Viola, Clara Limbaeck‐Stanic

**Affiliations:** ^1^ Department of Cellular Pathology, Northwest London Pathology, Charing Cross Hospital Imperial College Healthcare NHS Trust London UK; ^2^ Department of Neuroradiology, Charing Cross Hospital Imperial College Healthcare NHS Trust London UK; ^3^ Division of Surgery and Cancer, Faculty of Medicine Imperial College London UK; ^4^ Department of Cytogenetics Eurofins Biomnis Laboratory Lyon France; ^5^ Department of Neurology, Stroke and Neurosciences Directorate, Charing Cross Hospital Imperial College Healthcare NHS Trust London UK; ^6^ Division of Brain Sciences, Faculty of Medicine Imperial College London UK

## Abstract

This case report describes the cytological features of a rare tumour: diffuse leptomeningeal glioneuronal tumour. This case highlights the value of cerebrospinal fluid analysis when this type of tumour is suspected, both for aiding the preliminary morphological diagnosis and for enabling potential molecular testing.

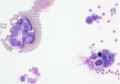

Diffuse leptomeningeal glioneuronal tumour (DLGNT), a recently recognised entity, is defined in the 2021 WHO Classification of Tumors of the Central Nervous System as a diffuse leptomeningeal disease which histologically demonstrates a monomorphic clear cell morphology, reminiscent of oligodendroglioma, commonly harbouring *BRAF* fusions as well as deletions of chromosome arm 1p.^1^ DLGNT is suspected radiologically by the presence of diffuse leptomeningeal disease; however, pathological diagnosis is necessary for diagnostic confirmation. This is challenging; tumour material from biopsies is typically sparse and initial biopsies are frequently non‐diagnostic.^2^ Cytological diagnosis via cerebrospinal fluid (CSF) is pragmatically more feasible; however, existing cases have largely failed to identify neoplastic cytology.^2^, ^3^, ^4^ We report a case of DLGNT in which CSF analysis determined the diagnosis and guided subsequent work‐up.

## 1 CASE HISTORY

**FIGURE 1:**
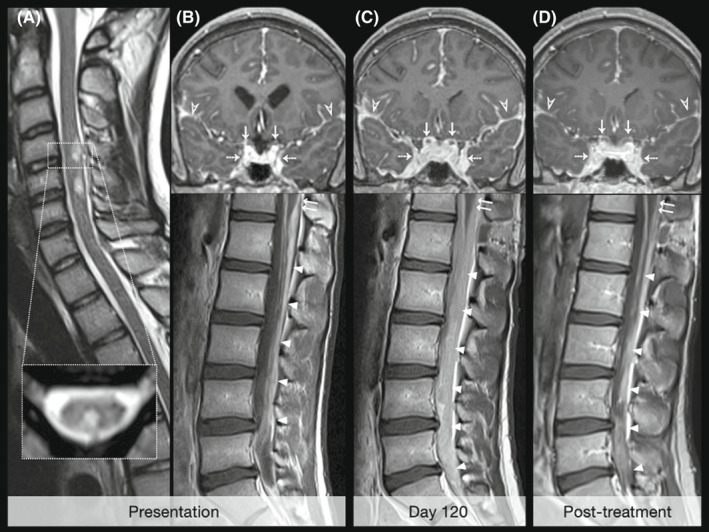
MRI features on initial presentation (A, B) and temporal evolution pre‐treatment (B, C) and post‐treatment (D). Small, T2 hyperintense pseudocystic lesions are present within the dorsal aspect of the cervical spinal cord on sagittal (A) and axial (A, inline) T2‐weighted images of the cervical spine. Coronal (B, top pane) post‐contrast T1‐weighted MR images of the brain demonstrate extensive nodular thickening and enhancement of the intracranial leptomeninges, including the Sylvian fissures (open arrowheads), basal cisterns, parasellar regions (dashed arrows), falx cerebri and middle cranial fossae. Suprasellar leptomeningeal disease encases the optic nerves on both sides (arrows). Serial coronal T1‐weighted images of the brain (B‐D, top row) and sagittal post‐contrast T1‐weighted images of the lumbosacral spine (B‐D, bottom row) obtained at initial presentation (B), 4 months after presentation (C) and 2 weeks following initiation of MEK‐inhibitor (D) demonstrate radiological progression over time (B, C) and subsequent response to treatment (D). On presentation, there was extensive intracranial leptomeningeal disease with associated communicating hydrocephalus (B, top pane). In the lumbosacral spine, leptomeningeal thickening and enhancement lining the conus medullaris (double arrows in B, bottom pane) and cauda equina (arrowheads in B, bottom pane) was evident. Four months later and following shunt insertion (C), the ventricles were decompressed but the leptomeningeal disease in the brain and spine had worsened (labels in C, top pane). Leptomeningeal thickening and enhancement lining the conus medullaris and cauda equina (double arrows and arrowheads in C, bottom pane) now completely fills the thecal sac and encases the neural structures. Two weeks after initiation of treatment (D), there was a significant improvement in the bulk of the intracranial (labels in D, top pane) and intraspinal enhancing leptomeningeal disease (labels in D, bottom pane).

A 19‐year‐old male presented to A&E in November 2020 with a 10‐day history of worsening morning headaches, nausea and vomiting. Initial computerised tomography (CT) of the head was normal. The patient self‐discharged prior to further work‐up but re‐presented in February 2021 with ongoing headaches. Fundoscopy now revealed bilateral papilloedema. CT demonstrated new mild hydrocephalus, and lumbar puncture showed a marginally raised opening pressure (28 cmH_2_O), elevated protein (0.8 g L^−1^) and mild pleocytosis (5 WBC cm^−3^). The patient again self‐discharged, but re‐presented in July 2021 with ongoing headaches and new blurred vision in the right eye. Bilateral papilloedema was again noted alongside diplopia on right lateral gaze and reduced visual acuity in the right eye. Lumbar puncture showed a markedly raised opening pressure (> 40 cmH_2_O) and a rising protein level (1.46 g L^−1^). Magnetic resonance imaging (MRI) revealed worsening hydrocephalus, diffuse cerebral and spinal leptomeningeal enhancement and non‐enhancing, cystic‐appearing lesions in the dorsal spinal cord (Figure 1A,B). A diagnosis of DLGNT was considered in addition to chronic inflammatory or infectious processes. Extensive bloodwork for infectious, inflammatory and neoplastic causes was non‐contributory. The patient was discharged on an empirical course of steroids, but re‐presented in August 2021 with worsening headaches; lumbar puncture showed a raised opening pressure (36 cmH_2_O). He underwent ventriculoperitoneal shunt insertion and interval MRI demonstrated improved hydrocephalus but progression of the leptomeningeal disease (Figure 1C).

## 2 MATERIALS AND METHODS

The patient underwent serial brain and dural biopsies (July 2021, September 2021, November 2021). CSF was obtained at the time of surgery for cytomorphological assessment. Tissue slides were examined using a variety of histochemical and immunohistochemical stains. Fluorescence in situ hybridisation (FISH) analysis was performed on the second biopsy. Further molecular profiling was performed on the third biopsy.

## 3 RESULTS

**FIGURE 2:**
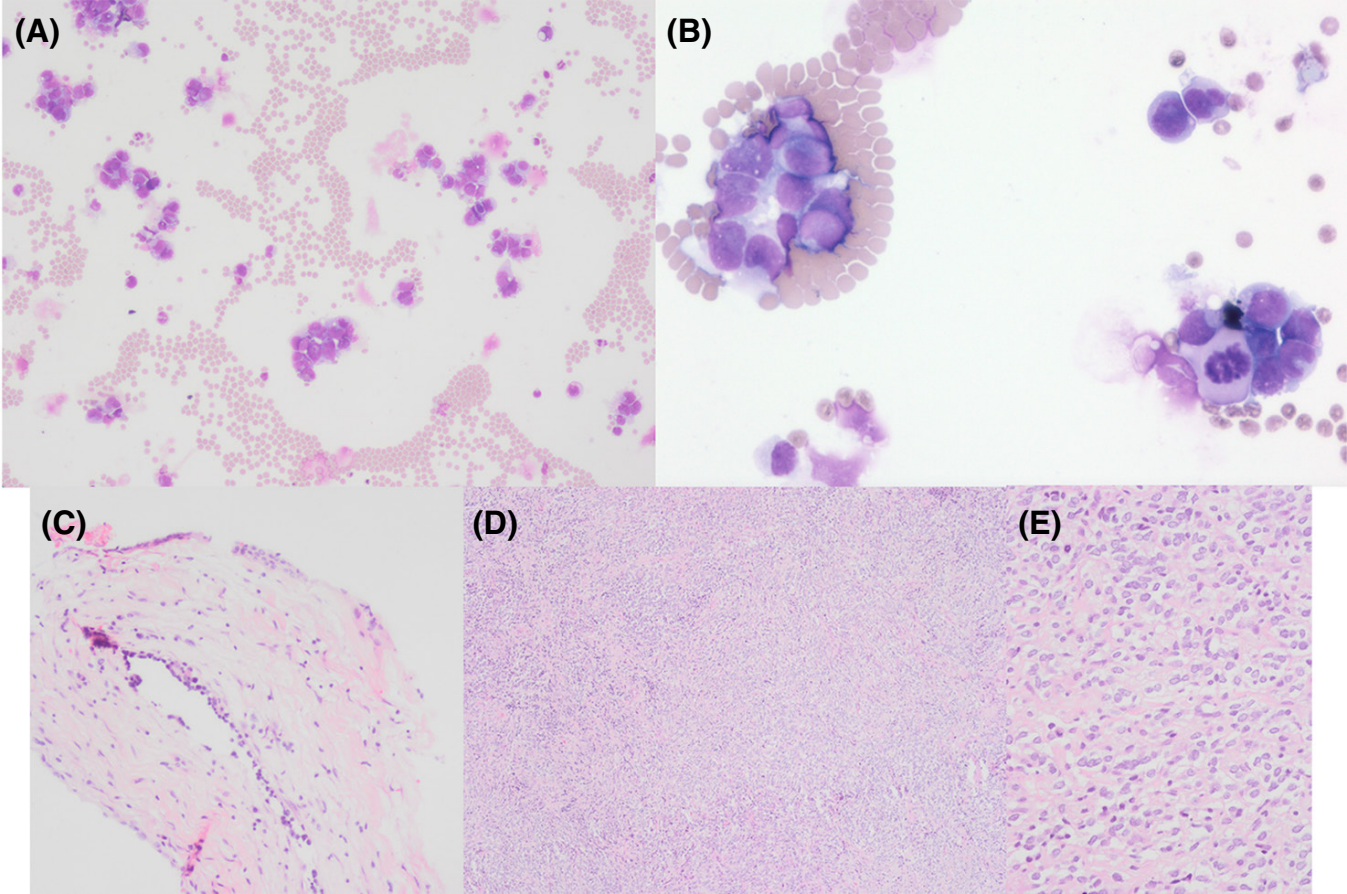
Serial cytological and histological findings of the leptomeningeal lesion. Cerebrospinal fluid analysis in September 2021 showed groups of, and singly dispersed, atypical epithelioid cells with irregular nuclear membranes, microvacuolated cytoplasm, pseudoinclusions, occasional nucleoli and mitoses (A, ×10 magnification; B, ×40 magnification). Biopsy at this time revealed focal infiltration of the meninges by mildly atypical glial cells with oligodroglial‐like morphology (C, ×20 magnification). Repeat biopsy (November 2021) showed meninges diffusely infiltrated by a cellular tumour (D, ×4 magnification), which at higher power is mitotically active with oligodendroglial‐like morphology (E, ×20 magnification)

The first biopsy (frontal and meningeal) showed no evidence of infection or neoplasia. Repeat dural biopsies were undertaken (September 2021). CSF obtained at this time revealed groups of, and singly dispersed, atypical epithelioid cells with irregular nuclear membranes, microvacuolated cytoplasm, pseudoinclusions, occasional nucleoli, and mitoses (Figure 2A,B). Immunocytochemistry on the cytospin preparation showed positive staining for S100 and negative staining for MNF116; additional staining or molecular tests could not be performed due to a lack of material. Histological examination revealed very focal infiltration of the meninges with bland small rounded cells (Figure 2C). Based on cytology findings, a small immunohistochemical panel was attempted, which showed that the cells expressed GFAP, S100 and Olig2 and were negative for MNF116, EMA, SMA and MelanA. The proliferation marker Ki67 labelled a few atypical nuclei. FISH revealed an isolated 1p deletion (with the Vysis LSI 1p36/1q25 and LSI 19q13/19p13 FISH Probe Kit). On this biopsy there was insufficient material for further molecular studies. Overall, the features were that of a neoplasm of glial/glioneuronal lineage and strongly suggested DLGNT.

A third biopsy (November 2021) showed meninges infiltrated with a highly cellular, mitotically active tumour with oligodendroglial‐like morphology (Figure 2D,E). Molecular profiling on this biopsy demonstrated a MAPK pathway alteration (KIAA1549‐BRAF fusion) on RNA‐based next generation sequencing, compatible with DLGNT. Methylation profiling using an Illumina EPIC array was performed (analysed using the DKFZ Heidelberg CNS tumour classifier). Methylation classifier results (bCHAPs, in‐house bioinformatic pipeline v6.0) classified the tumour as DLGNT, with a calibrated score of 0.7767 (low confidence). The research classifier (v12.3) classified the tumour as a DLGNT subtype 2 (DLGNT‐MC‐2), with a high degree of confidence (score 0.9987). A DNA sequencing panel detected no pertinent variants of unknown significance. Copy number analysis showed a complex karyotype with gains of chromosomes 1q, 2, 15, 17q and segmental gains of chromosomes 7q (BRAF) and 12q, and losses of chromosomes 1p, 4, 5, 8, 9 and 21. The patient was initiated on a MEK inhibitor and an interval MRI 1‐month later showed evidence of early treatment response (Figure 1D).

## 4 DISCUSSION

We report a case in which the clinical, radiological, and pathomolecular findings were consistent with DLGNT.^1^ Unusually, the initial diagnosis was based on cytology findings, guiding further work‐up. The differential diagnosis from clinical, imaging, and initial cytological assessment alone included, besides DLGNT, other primary neoplasia such as diffuse leptomeningeal gliomatosis and metastatic tumours involving meningeal structures. With further tests, the tumour showed the typical features of co‐expression of GFAP, S100 and Olig2, and a KIAA1549‐BRAF fusion alongside loss of chromosome 1p. Of note, the tumour showed high‐grade histopathological progression, whereas the majority of DLGNTs are low‐grade.^3^

Diffuse leptomeningeal glioneuronal tumour is rare, yet case reports have emerged: the largest case series of 36 patients was first reported in 2012 and ~100 cases have since been reported.^5^ CSF analysis typically reveals a mild hyperproteinaemia and lymphocyte pleocytosis, and is done to exclude an infectious aetiology; tumour cells are rarely identified.^3^, ^6^

To date only two other case reports of DLGNT have described neoplastic cells in the CSF.^5^, ^7^ In both, however, the supportive cytology findings emerged later in the disease, subsequent to histopathological diagnosis, and were therefore not critical in guiding work‐up. The first reported case was reported prior to the 2016 WHO classification and was therefore given the likely synonymous diagnosis of “primary diffuse leptomeningeal oligodendroglioma” but met the diagnostic criteria for DLGNT.^7^ Although initial CSF analysis revealed “no neoplastic cells”, CSF obtained therapeutically from the shunt reservoir was reported as “positive for neoplastic cells”, but no details of the cytomorphology are given.^7^ The second case, from a large DLGNT case series, reported: “neoplastic cells… present in CSF at later time points in the disease”; however, no further details were given regarding the CSF findings.^5^ Indeed, the authors highlight the fact that a histological diagnosis is usually required since neoplastic cells are rarely found in CSF.^5^

Diffuse leptomeningeal glioneuronal tumour is typically a low‐grade tumour; however, a subset of reports describe high‐grade progression, including frank anaplasia, elevated mitotic index, and necrosis.^5^, ^8^, ^9^ Our case demonstrated anaplastic progression; the third biopsy showed high‐grade morphology but with a molecular profile in keeping with DLGNT. Additionally, chromosome 1q gain was detected, which is known to be associated with aggressive behaviour and shorter survival, in keeping with DLGNT subtype 2 (DLGNT‐MCC‐2).^10^ We can hypothesise that the typical low‐grade indolent subset of DLGNTs results in a paucity of tumour cells within the CSF and therefore non‐diagnostic cytology. By contrast, the rarer high‐grade anaplastic subset may result in greater shedding of tumour cells within the CSF, and therefore greater diagnostic yield, as demonstrated here.

## 5 CONCLUSION

We present a case of DLGNT in which CSF analysis aided the diagnosis. DLGNT often poses a diagnostic challenge as imaging may be non‐specific and it can be difficult to acquire representative tissue at biopsy. We illustrate the importance of a persistent approach and consideration of repeat CSF testing when index of suspicion is high. In particular, this case highlights the value of CSF analysis in potentially reducing time to definitive diagnosis and appropriate treatment. Although diagnostic yield may be low, CSF is frequently obtained with relative ease at initial work‐up or therapeutically later in the disease. Hence, the opportunity for cytological CSF analysis should not be missed, both for aiding the preliminary morphological diagnosis and for enabling potential molecular testing.

## AUTHOR CONTRIBUTIONS

MPJ conducted the literature search and drafted the initial manuscript. EL and LD analysed the radiological findings. BQ conducted the molecular analysis. HJ was responsible for the clinical management of the patient. PV analysed the cytological findings. CL was responsible for the study concept, and analysed the histopathological and cytological findings. All authors edited and critically reviewed the manuscript.

## FUNDING INFORMATION

MPJ is funded by the National Institute of Health Research. PV is supported by BRC funding. The funding bodies had no role in the design of the study, nor in collection, analysis, or interpretation of data or in writing the manuscript.

## CONFLICT OF INTEREST

No conflicts of interest declared.

## PATIENT CONSENT

Written consent for publication was obtained from the patient.

## Data Availability

Data sharing not applicable to this article as no datasets were generated or analysed during the current study.
